# Diethyl [benzyl­amino­(1,3-diphenyl-1*H*-pyrazol-4-­yl)meth­yl]phospho­nate

**DOI:** 10.1107/S1600536811032776

**Published:** 2011-08-27

**Authors:** G. Jagadeesan, G. Suresh, B. Nandakumar, P. T. Perumal, S. Aravindhan

**Affiliations:** a Department of Physics, Presidency College, Chennai 600 005, India; b Organic Chemistry Laboratory, CLRI, Chennai 600 020, India

## Abstract

In the title compound, C_27_H_30_N_3_O_3_P, the pyrazole ring is essentially planar [maximum deviation = 0.002 (2) Å] and it forms dihedral angles of 9.3 (1) and 40.2 (1)°, respectively, with the phenyl rings attached to the N and C atoms. In the crystal, pairs of centrosymmetrically related mol­ecules are linked into dimers by N—H⋯O hydrogen bonds.

## Related literature

For the bioactivities of pyrazole derivatives, see: Sullivan *et al.* (2006 ▶); Patel *et al.* (2010 ▶); Siu *et al.* (2008 ▶).
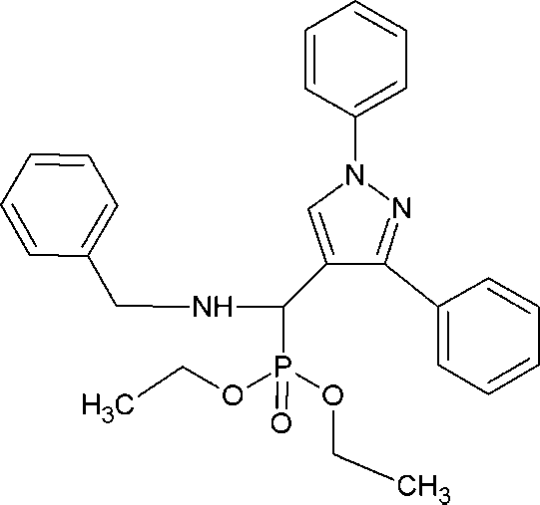


## Experimental

### 

#### Crystal data

C_27_H_30_N_3_O_3_P
*M* *_r_* = 475.51
Monoclinic, 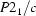

*a* = 10.9534 (4) Å
*b* = 9.3777 (3) Å
*c* = 25.0690 (8) Å
β = 101.233 (2)°
*V* = 2525.70 (15) Å^3^
*Z* = 4
Mo *K*α radiation
μ = 0.14 mm^−1^
*T* = 293 K
0.2 × 0.2 × 0.2 mm

#### Data collection

Oxford Diffraction Xcalibur-S diffractometer
Absorption correction: multi-scan (*CrysAlis PRO*; Oxford Diffraction, 2009 ▶) *T*_min_ = 0.980, *T*_max_ = 0.990
28506 measured reflections
6292 independent reflections
4220 reflections with *I* > 2σ(*I*)
*R*_int_ = 0.031

#### Refinement

*R*[*F* ^2^ > 2σ(*F* ^2^)] = 0.045
*wR*(*F* ^2^) = 0.124
*S* = 1.03
6292 reflections
403 parameters
H atoms treated by a mixture of independent and constrained refinement
Δρ_max_ = 0.33 e Å^−3^
Δρ_min_ = −0.31 e Å^−3^

### 

Data collection: *CrysAlis PRO* (Oxford Diffraction, 2009 ▶); cell refinement: *CrysAlis PRO*; data reduction: *CrysAlis PRO*; program(s) used to solve structure: *SHELXS97* (Sheldrick, 2008 ▶); program(s) used to refine structure: *SHELXL97* (Sheldrick, 2008 ▶); molecular graphics: *ORTEP-3* (Farrugia, 1997 ▶); software used to prepare material for publication: *PLATON* (Spek, 2009 ▶).

## Supplementary Material

Crystal structure: contains datablock(s) global, I. DOI: 10.1107/S1600536811032776/ci5195sup1.cif

Structure factors: contains datablock(s) I. DOI: 10.1107/S1600536811032776/ci5195Isup2.hkl

Supplementary material file. DOI: 10.1107/S1600536811032776/ci5195Isup3.cml

Additional supplementary materials:  crystallographic information; 3D view; checkCIF report

## Figures and Tables

**Table 1:** Hydrogen-bond geometry (Å, °)

*D*—H⋯*A*	*D*—H	H⋯*A*	*D*⋯*A*	*D*—H⋯*A*
N7—H7⋯O2^i^	0.89 (2)	2.16 (2)	2.9891 (19)	155 (2)

Symmetry code: (i) 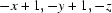.

**Fig. 1.**
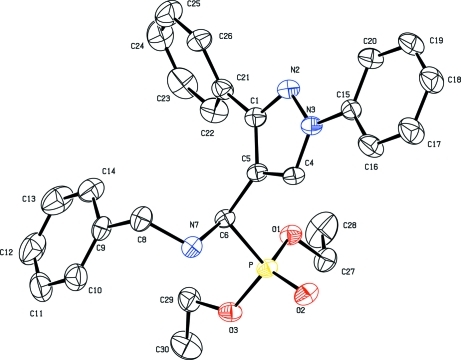
The molecular structure of the title compound, showing 30% probability displacement ellipsoids for non-H atoms. H atoms have been omitted for clarity.

**Fig. 2.**
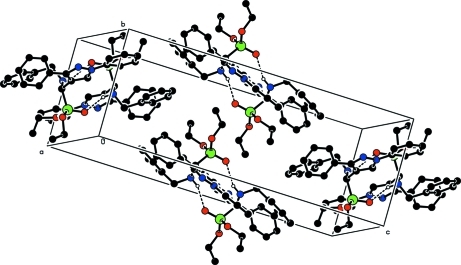
A view of the crystal packing. H atoms not involved in hydrogen bonding (dashed lines) have been omitted for clarity.

## supplementary crystallographic information

### Comment

Pyrazoles exhibit a variety of pharmacological properties for e.g antibacterial and anti-inflammatory activities (Sullivan *et al.*, 2006; Patel *et al.*, 2010). One of the pyrazole derivatives shows nucleosidase inhibitory activity against *Staphylococcus aureus* (Siu *et al.*, 2008). In view of their importance, the crystal structure determination of the title compound was carried out and the results are presented here.

The molecular structure of the title compound is shown in Fig. 1. The pyrazole ring is planar; the phenyl ring attached to N3 is almost coplanar [dihedral angle 9.3 (1)°] with it whereas the phenyl attached to C21 is tilted by 40.2 (1)°. Ester substitutions at the P atom lie anti to bulky substitutions at atom C6 [O1—P—C6—N7 = 169.2 (1)° and O3—P—C6—C5 = 175.2 (1)°].

In the crystal, pairs of centrosymmetrically related molecules are linked into dimers by N—H···O hydrogen bonds (Fig. 2).

### Experimental

A mixture of 3-diphenyl-1H-pyrazole-4-carbaldehyde (1 mmol), benzyl amine (1 mmol), diethyl phosphate (1.5 mmol) and pottasium hydrogen sulfate (20 mol%) under neat condition was stirred at room temperature. After completion of the reaction as indicated by TLC, it was poured into water and extracted with ethyl acetate. The organic layer was dried over sodium sulfate and concentrated under vacuum. The crude product was chromatographed. Single crystals were grown by slow evaporation an ethyl acetate-petroleum ether solution.

### Refinement

Atoms H23, H24, H25, H28A, H28B and H28C were positioned geometrically and refined using a riding model [C–H = 0.93 or 0.96 Å and U_iso_(H) = 1.2–1.5U_eq_(C)]. The remaining H atoms were located in a difference map and refined freely [N–H = 0.89 (2) Å and C–H = 0.90 (2)–1.03 (3) Å].

### Crystal data

**Table:**

C_27_H_30_N_3_O_3_P	*F*(000) = 1008
*M**_r_* = 475.51	*D*_x_ = 1.251 Mg m^−^^3^
Monoclinic, *P*2_1_/*c*	Mo *K*α radiation, λ = 0.71073 Å
Hall symbol: -P 2ybc	Cell parameters from 8725 reflections
*a* = 10.9534 (4) Å	θ = 2.8–29.1°
*b* = 9.3777 (3) Å	µ = 0.14 mm^−^^1^
*c* = 25.0690 (8) Å	*T* = 293 K
β = 101.233 (2)°	Block, colourless
*V* = 2525.70 (15) Å^3^	0.2 × 0.2 × 0.2 mm
*Z* = 4	

### Data collection

**Table:**

Oxford Diffraction Xcalibur-S diffractometer	6292 independent reflections
Radiation source: fine-focus sealed tube	4220 reflections with *I* > 2σ(*I*)
graphite	*R*_int_ = 0.031
Detector resolution: 15.9948 pixels mm^-1^	θ_max_ = 28.4°, θ_min_ = 2.3°
ω scans	*h* = −13→14
Absorption correction: multi-scan (*CrysAlis PRO*; Oxford Diffraction, 2009)	*k* = −12→12
*T*_min_ = 0.980, *T*_max_ = 0.990	*l* = −33→31
28506 measured reflections	

### Refinement

**Table:**

Refinement on *F*^2^	Primary atom site location: structure-invariant direct methods
Least-squares matrix: full	Secondary atom site location: difference Fourier map
*R*[*F*^2^ > 2σ(*F*^2^)] = 0.045	Hydrogen site location: inferred from neighbouring sites
*wR*(*F*^2^) = 0.124	H atoms treated by a mixture of independent and constrained refinement
*S* = 1.03	*w* = 1/[σ^2^(*F*_o_^2^) + (0.0515*P*)^2^ + 0.7236*P*] where *P* = (*F*_o_^2^ + 2*F*_c_^2^)/3
6292 reflections	(Δ/σ)_max_ = 0.001
403 parameters	Δρ_max_ = 0.33 e Å^−^^3^
0 restraints	Δρ_min_ = −0.31 e Å^−^^3^

### Special details

**Table:**

Geometry. All e.s.d.'s (except the e.s.d. in the dihedral angle between two l.s. planes) are estimated using the full covariance matrix. The cell e.s.d.'s are taken into account individually in the estimation of e.s.d.'s in distances, angles and torsion angles; correlations between e.s.d.'s in cell parameters are only used when they are defined by crystal symmetry. An approximate (isotropic) treatment of cell e.s.d.'s is used for estimating e.s.d.'s involving l.s. planes.
Refinement. Refinement of *F*^2^ against ALL reflections. The weighted *R*-factor *wR* and goodness of fit *S* are based on *F*^2^, conventional *R*-factors *R* are based on *F*, with *F* set to zero for negative *F*^2^. The threshold expression of *F*^2^ > σ(*F*^2^) is used only for calculating *R*-factors(gt) *etc*. and is not relevant to the choice of reflections for refinement. *R*-factors based on *F*^2^ are statistically about twice as large as those based on *F*, and *R*- factors based on ALL data will be even larger.

### Fractional atomic coordinates and isotropic or equivalent isotropic displacement parameters (Å^2^)

**Table:**

	*x*	*y*	*z*	*U*_iso_*/*U*_eq_	
H8B	0.4103 (19)	0.338 (2)	0.1155 (8)	0.066 (6)*	
H8A	0.549 (2)	0.373 (2)	0.1466 (8)	0.061 (6)*	
H7	0.4879 (18)	0.455 (2)	0.0558 (9)	0.057 (6)*	
H17	0.892 (3)	0.223 (3)	−0.0967 (12)	0.098 (9)*	
H4	0.6887 (17)	0.4658 (19)	0.0136 (8)	0.046 (5)*	
H29B	0.375 (2)	0.800 (3)	0.1338 (10)	0.080 (7)*	
H22	0.768 (2)	0.755 (2)	0.1823 (9)	0.073 (7)*	
H29A	0.497 (2)	0.891 (2)	0.1420 (9)	0.069 (7)*	
H19	1.200 (3)	0.240 (3)	0.0216 (10)	0.095 (9)*	
H20	1.085 (2)	0.343 (3)	0.0812 (10)	0.078 (7)*	
H14	0.572 (2)	0.522 (3)	0.2261 (10)	0.085 (8)*	
H16	0.779 (2)	0.327 (3)	−0.0394 (9)	0.077 (7)*	
H10	0.244 (2)	0.501 (3)	0.1297 (10)	0.071 (7)*	
H11	0.156 (3)	0.632 (3)	0.1919 (11)	0.103 (10)*	
H26	0.930 (2)	0.371 (3)	0.2181 (9)	0.079 (7)*	
H18	1.105 (3)	0.180 (3)	−0.0676 (11)	0.096 (8)*	
H6	0.5905 (15)	0.6388 (17)	0.1330 (7)	0.036 (4)*	
H30A	0.321 (3)	1.032 (3)	0.1460 (13)	0.110 (10)*	
H30B	0.252 (3)	0.988 (3)	0.0857 (12)	0.111 (10)*	
H27B	0.715 (2)	1.005 (3)	0.0291 (10)	0.087 (8)*	
H27A	0.584 (3)	1.038 (3)	0.0488 (11)	0.102 (9)*	
H13	0.494 (3)	0.653 (3)	0.2873 (13)	0.113 (11)*	
H30C	0.379 (4)	1.079 (5)	0.0955 (17)	0.168 (18)*	
H12	0.282 (3)	0.710 (4)	0.2731 (15)	0.146 (13)*	
P	0.55474 (4)	0.75862 (4)	0.054083 (18)	0.03871 (13)	
O1	0.66355 (11)	0.86067 (11)	0.08051 (5)	0.0448 (3)	
N7	0.47930 (14)	0.50807 (15)	0.08404 (6)	0.0428 (3)	
N3	0.85342 (13)	0.41796 (14)	0.06060 (6)	0.0420 (3)	
O3	0.43152 (11)	0.82882 (13)	0.06482 (5)	0.0491 (3)	
N2	0.90518 (14)	0.44221 (15)	0.11333 (6)	0.0451 (4)	
O2	0.54283 (13)	0.73175 (13)	−0.00419 (5)	0.0526 (3)	
C6	0.58773 (16)	0.60281 (16)	0.09730 (7)	0.0376 (4)	
C5	0.71061 (16)	0.53605 (16)	0.09294 (7)	0.0392 (4)	
C1	0.81900 (16)	0.51432 (17)	0.13319 (7)	0.0427 (4)	
C4	0.73742 (17)	0.47270 (17)	0.04735 (8)	0.0426 (4)	
C15	0.92211 (17)	0.34705 (17)	0.02579 (7)	0.0436 (4)	
C21	0.84419 (17)	0.5557 (2)	0.19091 (8)	0.0478 (4)	
C8	0.4667 (2)	0.41576 (19)	0.12999 (9)	0.0541 (5)	
C9	0.4121 (2)	0.49577 (19)	0.17170 (8)	0.0512 (5)	
C20	1.0464 (2)	0.3187 (2)	0.04401 (10)	0.0602 (5)	
C29	0.4167 (2)	0.8764 (2)	0.11825 (10)	0.0604 (5)	
C27	0.6709 (2)	1.0066 (2)	0.06104 (10)	0.0580 (5)	
C26	0.9060 (2)	0.4626 (3)	0.23015 (9)	0.0628 (6)	
C16	0.8658 (2)	0.3111 (3)	−0.02600 (9)	0.0652 (6)	
C14	0.4848 (3)	0.5419 (3)	0.21966 (9)	0.0682 (6)	
C23	0.8325 (3)	0.7229 (3)	0.26239 (12)	0.0854 (8)	
H23	0.8065	0.8106	0.2734	0.102*	
C22	0.8087 (2)	0.6875 (3)	0.20769 (10)	0.0657 (6)	
C10	0.2871 (3)	0.5309 (3)	0.16212 (11)	0.0697 (6)	
C18	1.0589 (2)	0.2218 (3)	−0.04303 (12)	0.0723 (7)	
C17	0.9346 (2)	0.2490 (3)	−0.06048 (11)	0.0760 (7)	
C12	0.3125 (4)	0.6572 (4)	0.24570 (13)	0.0964 (10)	
C11	0.2376 (3)	0.6101 (3)	0.19938 (15)	0.0875 (9)	
C24	0.8937 (3)	0.6297 (4)	0.30026 (11)	0.0899 (9)	
H24	0.9102	0.6545	0.3369	0.108*	
C19	1.1140 (2)	0.2559 (3)	0.00886 (12)	0.0724 (7)	
C28	0.7401 (3)	1.0934 (3)	0.10496 (12)	0.1023 (10)	
H28C	0.7453	1.1896	0.0925	0.153*	
H28B	0.8225	1.0552	0.1161	0.153*	
H28A	0.6982	1.0925	0.1352	0.153*	
C30	0.3365 (3)	1.0064 (3)	0.11075 (16)	0.0873 (9)	
C13	0.4355 (4)	0.6236 (3)	0.25610 (12)	0.0949 (9)	
C25	0.9306 (3)	0.5004 (4)	0.28426 (10)	0.0862 (8)	
H25	0.9726	0.4372	0.3101	0.103*	

### Atomic displacement parameters (Å^2^)

**Table:**

	*U*^11^	*U*^22^	*U*^33^	*U*^12^	*U*^13^	*U*^23^
P	0.0444 (3)	0.0310 (2)	0.0393 (2)	0.00695 (17)	0.00468 (18)	−0.00017 (16)
O1	0.0494 (7)	0.0322 (5)	0.0508 (7)	0.0011 (5)	0.0044 (6)	0.0044 (5)
N7	0.0512 (9)	0.0361 (7)	0.0410 (8)	−0.0005 (6)	0.0088 (7)	−0.0030 (6)
N3	0.0411 (8)	0.0384 (7)	0.0450 (8)	0.0053 (6)	0.0044 (6)	0.0006 (6)
O3	0.0474 (8)	0.0431 (6)	0.0550 (8)	0.0123 (5)	0.0056 (6)	−0.0009 (5)
N2	0.0442 (9)	0.0438 (8)	0.0440 (9)	0.0033 (6)	0.0004 (7)	0.0002 (6)
O2	0.0710 (9)	0.0441 (7)	0.0408 (7)	0.0096 (6)	0.0065 (6)	−0.0016 (5)
C6	0.0439 (10)	0.0321 (7)	0.0357 (9)	0.0042 (6)	0.0048 (7)	−0.0011 (6)
C5	0.0424 (10)	0.0317 (7)	0.0418 (9)	0.0036 (6)	0.0037 (7)	0.0025 (6)
C1	0.0443 (10)	0.0348 (8)	0.0469 (10)	−0.0003 (7)	0.0040 (8)	−0.0002 (7)
C4	0.0426 (10)	0.0398 (8)	0.0427 (10)	0.0076 (7)	0.0017 (8)	0.0021 (7)
C15	0.0429 (10)	0.0368 (8)	0.0523 (11)	0.0032 (7)	0.0121 (8)	0.0028 (7)
C21	0.0404 (10)	0.0528 (10)	0.0485 (11)	−0.0056 (8)	0.0040 (8)	−0.0055 (8)
C8	0.0726 (15)	0.0342 (9)	0.0572 (12)	−0.0024 (9)	0.0168 (11)	0.0030 (8)
C9	0.0660 (13)	0.0407 (9)	0.0493 (11)	−0.0048 (8)	0.0173 (10)	0.0080 (8)
C20	0.0493 (13)	0.0622 (12)	0.0670 (15)	0.0113 (10)	0.0058 (11)	0.0011 (10)
C29	0.0654 (15)	0.0579 (12)	0.0633 (14)	0.0136 (11)	0.0261 (12)	0.0002 (10)
C27	0.0689 (15)	0.0381 (9)	0.0672 (14)	−0.0022 (9)	0.0138 (12)	0.0137 (9)
C26	0.0624 (14)	0.0729 (15)	0.0489 (13)	0.0015 (11)	0.0004 (10)	0.0021 (10)
C16	0.0445 (13)	0.0882 (16)	0.0628 (14)	0.0068 (11)	0.0101 (10)	−0.0175 (12)
C14	0.0804 (18)	0.0683 (14)	0.0539 (14)	0.0128 (12)	0.0081 (12)	0.0023 (10)
C23	0.0794 (18)	0.0889 (18)	0.087 (2)	−0.0169 (14)	0.0155 (15)	−0.0439 (16)
C22	0.0662 (15)	0.0578 (12)	0.0681 (15)	−0.0067 (11)	0.0014 (11)	−0.0192 (11)
C10	0.0709 (17)	0.0727 (15)	0.0675 (16)	−0.0130 (12)	0.0184 (13)	0.0042 (12)
C18	0.0666 (16)	0.0752 (15)	0.0825 (18)	0.0156 (12)	0.0326 (14)	−0.0035 (12)
C17	0.0630 (16)	0.0997 (19)	0.0689 (16)	0.0079 (13)	0.0214 (13)	−0.0199 (14)
C12	0.138 (3)	0.095 (2)	0.0654 (19)	0.030 (2)	0.044 (2)	0.0089 (15)
C11	0.079 (2)	0.0930 (19)	0.103 (2)	0.0139 (16)	0.0481 (19)	0.0228 (17)
C24	0.089 (2)	0.128 (3)	0.0521 (15)	−0.0296 (18)	0.0126 (14)	−0.0301 (16)
C19	0.0474 (14)	0.0824 (16)	0.0898 (19)	0.0193 (12)	0.0191 (13)	0.0060 (13)
C28	0.155 (3)	0.0493 (13)	0.099 (2)	−0.0336 (16)	0.0141 (19)	−0.0041 (13)
C30	0.085 (2)	0.0713 (17)	0.109 (2)	0.0256 (15)	0.028 (2)	−0.0209 (16)
C13	0.130 (3)	0.094 (2)	0.0570 (17)	0.0257 (19)	0.0100 (17)	−0.0089 (14)
C25	0.0901 (19)	0.114 (2)	0.0484 (14)	−0.0115 (16)	−0.0018 (13)	0.0037 (14)

### Geometric parameters (Å, °)

**Table:**

P—O2	1.4629 (13)	C27—H27B	1.02 (3)
P—O1	1.5708 (12)	C27—H27A	0.98 (3)
P—O3	1.5713 (13)	C26—C25	1.377 (3)
P—C6	1.8129 (16)	C26—H26	0.97 (3)
O1—C27	1.460 (2)	C16—C17	1.381 (3)
N7—C6	1.468 (2)	C16—H16	0.96 (2)
N7—C8	1.469 (2)	C14—C13	1.381 (4)
N7—H7	0.89 (2)	C14—H14	0.95 (3)
N3—C4	1.350 (2)	C23—C24	1.366 (4)
N3—N2	1.352 (2)	C23—C22	1.385 (3)
N3—C15	1.423 (2)	C23—H23	0.93
O3—C29	1.451 (2)	C22—H22	0.94 (2)
N2—C1	1.334 (2)	C10—C11	1.384 (4)
C6—C5	1.508 (2)	C10—H10	0.90 (2)
C6—H6	0.952 (17)	C18—C19	1.361 (4)
C5—C4	1.370 (2)	C18—C17	1.370 (4)
C5—C1	1.415 (2)	C18—H18	0.95 (3)
C1—C21	1.472 (3)	C17—H17	0.97 (3)
C4—H4	0.911 (18)	C12—C11	1.359 (5)
C15—C16	1.367 (3)	C12—C13	1.359 (5)
C15—C20	1.375 (3)	C12—H12	0.96 (4)
C21—C22	1.385 (3)	C11—H11	0.90 (3)
C21—C26	1.389 (3)	C24—C25	1.363 (4)
C8—C9	1.502 (3)	C24—H24	0.93
C8—H8B	0.98 (2)	C19—H19	0.94 (3)
C8—H8A	1.00 (2)	C28—H28C	0.96
C9—C14	1.376 (3)	C28—H28B	0.96
C9—C10	1.384 (3)	C28—H28A	0.96
C20—C19	1.387 (3)	C30—H30A	0.96 (3)
C20—H20	0.97 (2)	C30—H30B	1.03 (3)
C29—C30	1.493 (3)	C30—H30C	0.94 (5)
C29—H29B	0.97 (3)	C13—H13	0.95 (3)
C29—H29A	0.97 (2)	C25—H25	0.93
C27—C28	1.458 (3)		
			
O2—P—O1	116.02 (8)	C28—C27—H27A	113.5 (17)
O2—P—O3	109.31 (7)	O1—C27—H27A	105.6 (17)
O1—P—O3	106.20 (7)	H27B—C27—H27A	110 (2)
O2—P—C6	115.25 (7)	C25—C26—C21	120.7 (2)
O1—P—C6	101.10 (7)	C25—C26—H26	121.6 (14)
O3—P—C6	108.25 (8)	C21—C26—H26	117.7 (14)
C27—O1—P	121.11 (13)	C15—C16—C17	119.9 (2)
C6—N7—C8	111.99 (15)	C15—C16—H16	122.0 (14)
C6—N7—H7	108.4 (13)	C17—C16—H16	118.1 (14)
C8—N7—H7	109.5 (13)	C9—C14—C13	121.2 (3)
C4—N3—N2	111.81 (14)	C9—C14—H14	118.3 (15)
C4—N3—C15	127.68 (15)	C13—C14—H14	120.3 (15)
N2—N3—C15	120.46 (14)	C24—C23—C22	120.5 (3)
C29—O3—P	122.76 (13)	C24—C23—H23	119.8
C1—N2—N3	104.94 (14)	C22—C23—H23	119.8
N7—C6—C5	115.25 (13)	C23—C22—C21	120.3 (2)
N7—C6—P	107.10 (11)	C23—C22—H22	118.7 (14)
C5—C6—P	111.61 (12)	C21—C22—H22	121.0 (14)
N7—C6—H6	107.7 (10)	C9—C10—C11	121.0 (3)
C5—C6—H6	110.5 (10)	C9—C10—H10	114.4 (16)
P—C6—H6	104.0 (10)	C11—C10—H10	124.5 (16)
C4—C5—C1	104.24 (15)	C19—C18—C17	119.3 (2)
C4—C5—C6	125.46 (15)	C19—C18—H18	121.5 (16)
C1—C5—C6	130.17 (15)	C17—C18—H18	119.2 (16)
N2—C1—C5	111.30 (15)	C18—C17—C16	120.5 (3)
N2—C1—C21	119.35 (16)	C18—C17—H17	121.1 (17)
C5—C1—C21	129.32 (16)	C16—C17—H17	118.3 (17)
N3—C4—C5	107.71 (16)	C11—C12—C13	120.0 (3)
N3—C4—H4	123.6 (12)	C11—C12—H12	123 (2)
C5—C4—H4	128.7 (12)	C13—C12—H12	117 (2)
C16—C15—C20	120.14 (19)	C12—C11—C10	120.0 (3)
C16—C15—N3	120.21 (17)	C12—C11—H11	120.9 (19)
C20—C15—N3	119.61 (17)	C10—C11—H11	119.0 (19)
C22—C21—C26	118.2 (2)	C25—C24—C23	119.9 (2)
C22—C21—C1	121.60 (18)	C25—C24—H24	120.1
C26—C21—C1	120.17 (18)	C23—C24—H24	120.1
N7—C8—C9	111.25 (15)	C18—C19—C20	121.0 (2)
N7—C8—H8B	107.3 (12)	C18—C19—H19	120.9 (16)
C9—C8—H8B	108.7 (12)	C20—C19—H19	118.1 (16)
N7—C8—H8A	110.5 (12)	C27—C28—H28C	109.5
C9—C8—H8A	111.1 (12)	C27—C28—H28B	109.5
H8B—C8—H8A	107.8 (17)	H28C—C28—H28B	109.5
C14—C9—C10	117.5 (2)	C27—C28—H28A	109.5
C14—C9—C8	121.6 (2)	H28C—C28—H28A	109.5
C10—C9—C8	120.8 (2)	H28B—C28—H28A	109.5
C15—C20—C19	119.1 (2)	C29—C30—H30A	106.8 (19)
C15—C20—H20	119.4 (14)	C29—C30—H30B	112.3 (17)
C19—C20—H20	121.5 (14)	H30A—C30—H30B	108 (2)
O3—C29—C30	107.7 (2)	C29—C30—H30C	109 (3)
O3—C29—H29B	106.8 (14)	H30A—C30—H30C	112 (3)
C30—C29—H29B	109.8 (15)	H30B—C30—H30C	109 (3)
O3—C29—H29A	111.0 (13)	C12—C13—C14	120.2 (3)
C30—C29—H29A	113.8 (14)	C12—C13—H13	125.0 (19)
H29B—C29—H29A	108 (2)	C14—C13—H13	115 (2)
C28—C27—O1	108.98 (18)	C24—C25—C26	120.4 (3)
C28—C27—H27B	110.2 (14)	C24—C25—H25	119.8
O1—C27—H27B	108.5 (14)	C26—C25—H25	119.8

### Hydrogen-bond geometry (Å, °)

**Table:**

*D*—H···*A*	*D*—H	H···*A*	*D*···*A*	*D*—H···*A*
N7—H7···O2^i^	0.89 (2)	2.16 (2)	2.9891 (19)	155 (2)

Symmetry codes: (i) −*x*+1, −*y*+1, −*z*.
